# An investigation of 2-hydrazinyl-1,3-benzothiazole, 4-amino-4H-1,2,4-triazole and 2-hydrazinyl-1H-benzimidazole applicability as derivatization reagents for HPLC-HRMS determination of steroid hormones in urine

**DOI:** 10.3389/fchem.2026.1836243

**Published:** 2026-08-07

**Authors:** Maria Zorina, Yu-Qi Feng, Sanka N. Atapattu, Victor Dotsenko, Vyacheslav Kindop, Mikhail Afonin, Quan-Fei Zhu, Azamat Temerdashev

**Affiliations:** 1 Department of Analytical Chemistry, Kuban State University, Krasnodar, Russia; 2 Textile University, Wuhan, China; 3 CanAm Bioresearch Inc., Winnipeg, MB, Canada; 4 Department of Organic Chemistry and Technology, Kuban State University, Krasnodar, Russia; 5 North Caucasus Federal University, Stavropol, Russia; 6 Sirius University of Science and Technology, Sirius Federal Territory, Sochi, Russia

**Keywords:** 2-hydrazinyl-1,3-benzothiazole, 2-hydrazinyl-1H-benzimidazole, 4-amino-4H-1,2,4-triazole, derivatization, HPLC-HRMS, steroid hormones, urine

## Abstract

2-hydrazinyl-1,3-benzothiazole (HBT), 4-amino-4H-1,2,4-triazole (AT) and 2-hydrazinyl-1H-benzimidazole (HBI) were evaluated in this study as novel derivatization agents for the determination of steroid hormones in urine. The derivatization reactions were based on the interaction between the carbonyl group of steroids and the primary amino group of novel reagents. Optimal conditions for the derivatization of steroid hormones with novel agents, including pH, agent concentration, temperature and time of reaction were established. The resulting derivatives were separated and detected using reversed-phase ultra-high performance liquid chromatography–quadrupole time-of-flight mass spectrometry. The methods developed for achieved detection limits of 0.015–0.15 ng/mL for HBT derivatives, 0.15 ng/mL for AT derivatives and 0.075–0.15 ng/mL for HBI derivatives, respectively. During the reaction with these new agents, both syn- and anti-forms of derivatives of steroid hormones are formed, similar to other hydrazines. Based on the molecular properties of the obtained derivatives, the influence of substituents on the chromatographic characteristics was studied. The procedure was successfully applied to the analysis of authentic urine samples, and the results were compared with those obtained from hydroxylamine-based and Girard’s reagent T-based derivatization, demonstrating the prospects for further study of new reagents.

## 1 Introduction

Modern analytical equipment provides analytical chemists with enormous opportunities for qualitative and quantitative analysis, but the determination of biogenic compounds which are found in biological fluids at low concentrations remains a complex task due to the need for multi-stage sample preparation, including preliminary purification, sample concentration and, in some cases, derivatization. Such compounds include steroid hormones, which play a crucial role in the human body (Stillwell, 2016). These compounds are of great interest to clinical diagnostics of endocrine, neurodegenerative and psychiatric disorders, such as polycystic ovarian syndrome (Shaaban et al., 2019), Cushing’s syndrome (Reincke and Fleseriu, 2023), Alzheimer’s disease (Vest and Pike, 2013) and depression (Pillerová et al., 2022). For clinical diagnostics, determining the steroid profile is necessary because, compared to measuring a single steroid hormone, it provides a more comprehensive view of the disease and allows for more accurate information to support the correct diagnosis (Yuan, 2022). Several biological matrices are used to determine the steroid profile. Despite the invasive sampling, serum and plasma are widely used for determination of steroid hormones (Gomez-Gomez et al., 2020). Dried blood spots are used for neonatal screening for congenital adrenal hyperplasia (Janzen et al., 2008). Analysis of urine is also commonly used in clinical diagnostics of steroid-related diseases (Taylor, 2013). Compared with blood, urine is a more convenient biological matrix because its non-invasive sampling. For steroid profiling there are alternative matrices and their analysis can expand clinical diagnostic capabilities, e.g., the analysis of amniotic fluid can help to obtain information about fetal development dynamics (Liu et al., 2020). Considering the low concentration levels of steroid hormones in human biological fluids, procedures for sample concentration are required. For this purpose, liquid-liquid extraction with diethyl or methyl tert-butyl ethers (Dmitrieva and Temerdashev, 2022; Temerdashev et al., 2022), solid-phase extraction with hydrophobic and hydrophilic-lipophilic balanced sorbents (Koskivuori et al., 2022; Taves et al., 2011) and dispersive liquid-liquid microextraction (Dmitrieva et al., 2020; Rezaee, 2023) are widely used. In addition, since steroid hormones are found in urine predominantly in conjugated forms, deconjugation is required before extraction. This procedure is usually conducted using enzymatic hydrolysis with β-glucuronidase from *E. coli* or β-glucuronidase and aryl sulfatase from *Helix pomatia* (Temerdashev et al., 2021).

For clinical diagnosis of steroid-related diseases, HPLC-MS is the preferred method since it enables the determination of conjugated steroid hormones without requiring hydrolysis and derivatization steps (Kaabia et al., 2018). Numerous clinical laboratories utilize reversed phase separation techniques in combination with electrospray ionization mode (Janzen et al., 2011). In addition, high-performance liquid chromatography coupled to high-resolution mass spectrometry (HPLC-HRMS) holds great promise for research purposes as it has emerged as a powerful tool for screening of various biogenic substances in biological fluids (Rochat, 2016). Unfortunately, HPLC-MS determination of steroid hormones from different classes is difficult due to significantly higher ionization efficiency in negative ion mode for many estrogens and several corticosteroids. Derivatization can be employed as a solution to this issue by generating derivatives that can be effectively ionized in positive ion mode while enhancing sensitivity (Marcos and Pozo, 2015). For the derivatization of steroid hormones containing a carbonyl group, various reagents with a primary amino group are used. They include hydroxylamine (HA), 2-hydrazinopyridine, 2-hydrazino-1-methylpyridine, Girard’s reagents, etc. The reactions with these derivatization agents lead to the formation of oximes and hydrazones of steroid hormones, which are capable of being effectively ionized in the positive ion mode. This can significantly enhance the sensitivity of ketosteroids determination by up to 1,000 times (Sun et al., 2021). The examples of determination of ketosteroids in biological fluids, including the derivatization step, are presented in Table 1.

**TABLE 1:** LC-MS determination of steroid hormones in biological matrices.

Analyte	Matrix	Derivatization reagent	Method	LOD	LOQ	References
Testosterone Dihydrotestosterone	Serum Prostate tissue	Picolinic acid	LC-MS/MS (ESI+)	—	5 pg/mL 0.2 pg/mg, 0.3 pg/mg	Yamashita et al. (2009)
Testosterone	Serum	Hydroxylamine	LC-MS/MS (ESI+)	0.5 ng/dL	1 ng/dL	Kushnir et al. (2006)
Testosterone Dihydrotestosterone Dehydroepiandrosterone Androstenone 4-androstenedione Cortisone 21-deoxycortisol Corticosterone Progesterone Estrone	Serum	Hydroxylamine	LC-MS/MS (ESI+)	—	0.05–5 ng/mL	Liu et al. (2019)
17α-hydroxyprogesterone	Saliva	2-hydrazinopyridine	LC-MS/MS (ESI+)	—	5 pg/mL	Shibayama et al. (2008)
Testosterone Dehydroepiandrosterone Cortisone Cortisol Progesterone 17α-hydroxyprogesterone	Saliva	2-hydrazinopyridine	LC-MS/MS (ESI+)	1.7 pg/mL– 0.02 ng/mL	5 pg/mL–0.07 ng/mL	Nadarajah et al. (2017)
Testosterone Dehydroepiandrosterone	Saliva	2-hydrazino-1-methylpyridine	LC-MS/MS (ESI+)	—	10 pg/mL	Shibayama et al. (2009)
17α-hydroxyprogesterone	Dried blood spots	Girard reagent P	LC-MS/MS (ESI+)	10 ng/mL	—	Lai et al. (2001)
Testosterone Dihydrotestosterone Cortisone Cortisol Corticosterone Estrone Progesterone 11α-hydroxyprogesterone Methyltestosterone	Urine	HBT; HBI; AT	LC-MS/MS (ESI+)	HBT: 0.015–0.15 ng/mL HBI: 0.075–0.15 ng/mL AT: 0.15 ng/mL	HBT: 0.030–0.15 ng/mL HBI: 0.15–0.3 ng/mL AT: 0.30 ng/mL	This research

**FIGURE 1:**
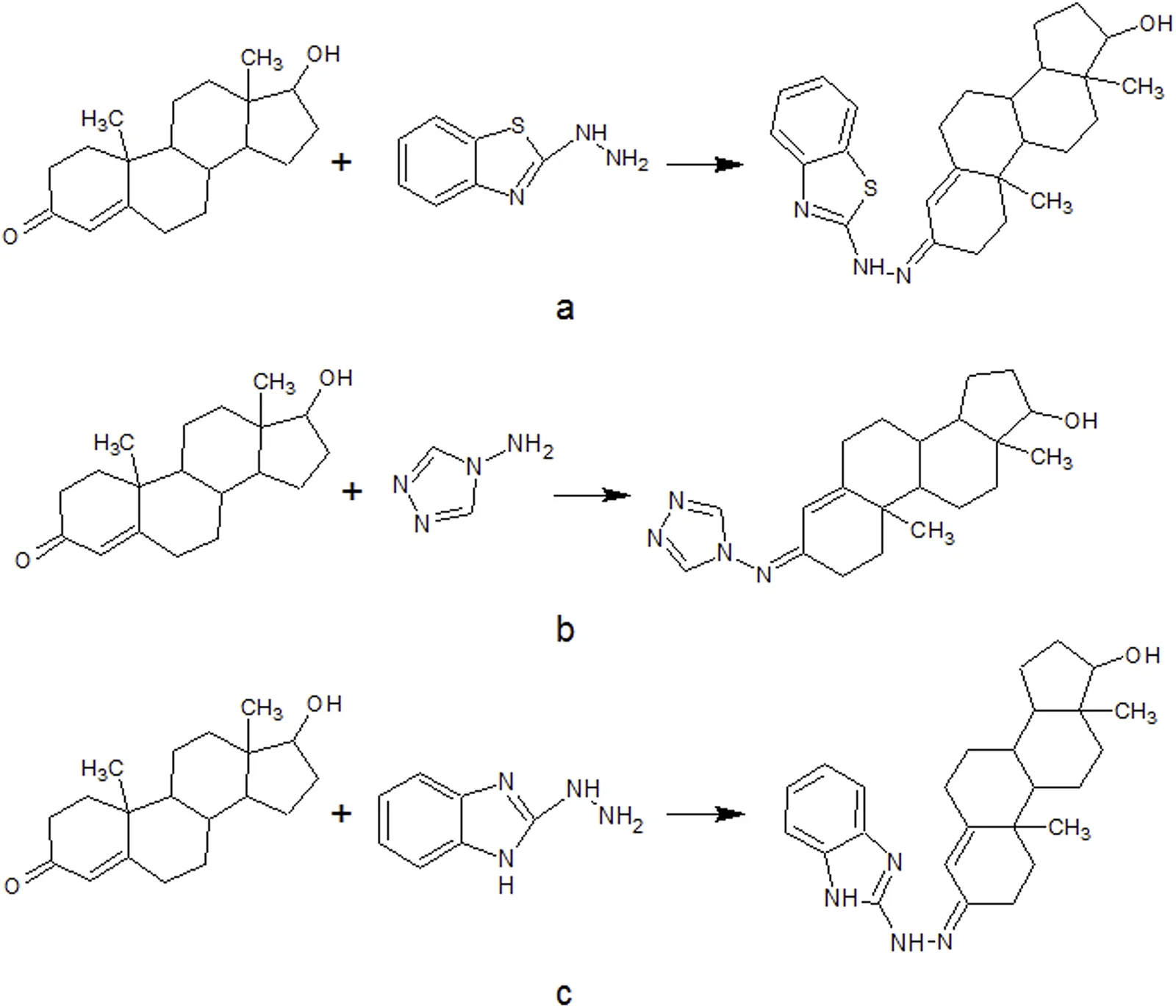
Schemes of testosterone derivatization with: **(a)**—HBT; **(b)**—AT; **(c)**—HBI.

Despite their advantages, these well-known reagents have several drawbacks, for example, the inability to derivatize steroid hormones from different classes due to steric hindrances and formation of syn- and anti-isomers. Therefore, there is a necessity for the use of novel reagents capable of overcoming these existing limitations. The factors, which should be considered before the use of new derivatization agents, include: functional group reactivity, derivative stability, artifact formation, chromatographic retention and detection behavior, molecular properties (acidity/basicity, solubility or polarity), analytical performance (detection and quantitation limits), application areas and ease of synthesis (David et al., 2021). Various commercially available heterocyclic compounds, which are widely used in organic synthesis, are promising candidates for potential derivatization agents. It is known that derivatives of benzothiazole, benzimidazole and 1,2,4-triazole are biologically active compounds which possess antimicrobial, fungicidal, antiviral, antitumor action (Mohapatra and Ganguly, 2024; Ovsyannikova et al., 2016; Yadav et al., 2023). Such derivatives include HBT, AT and HBI. Due to the presence of a primary amino group in their structure, these compounds can be used for derivatization of ketosteroids. The derivatization schemes are shown in Figure 1.

The objective of the present study was to investigate the suitability of novel reagents for the derivatization of steroid hormones in urine, enabling their subsequent determination by HPLC-HRMS. Additionally, we aimed to compare the efficacy of these new reagents with hydroxylamine (HA) and Girard’s reagent T (GRT), a commonly employed derivatization agent for steroid analysis using HPLC-MS methods. Optimization of various derivatization conditions was performed through a combination of multiple approaches. The influence of substituents on the chromatographic characteristics of the obtained derivatives was considered.

Developing derivatizing reagents of various classes is a separate, rather complex task, requiring an understanding of the limitations and capabilities of various functional groups. The simplest method is the production of oximes, which is widely used in modern practice; however, this method does not address all the challenges. Therefore, the use of reagents based on hydrazines and hydrazones is appropriate for a wide range of applications. They provide high sensitivity and, at the same time, significantly alter the physicochemical parameters of analytes that affect retention. This can also be used in analytical practice to combat matrix effects during quantitative analysis (Feng et al., 2025; Liu et al., 2011; Mandhane et al., 2015; Peng et al., 2024; Qin et al., 2025; Xiao et al., 2021).

## 2 Materials and methods

### 2.1 Chemicals and reagents

Standards of testosterone, dihydrotestosterone, cortisone, cortisol, corticosterone, estrone, progesterone, 11α-OH-progesterone, methyltestosterone (internal standard, IS) were purchased from Sigma-Aldrich (St. Lois, MO, USA). β-glucuronidase (Type H-2, ≥85.000 units/mL) from *H. pomatia* were obtained from Macklin Biochemical (Shanghai, China). HPLC grade acetonitrile (“Biosolve”, Jerusalem, Israel), 18.2 MΩ water (Milli-Q, Millipore, Molsheim, France), and formic acid (98%, Acros Organics, Geel, Belgium) were used as the mobile phase. HPLC grade methanol was purchased from Vecton (Saint-Petersburg, Russia). Potassium carbonate, potassium bicarbonate, sodium hydroxide, sodium tetraborate, sodium hydrogen phosphate, potassium dihydrogen phosphate, ammonium acetate (all of 99% purity) were obtained from Vecton (St. Petersburg, Russia).

### 2.2 Preparation of solutions

Stock standard solutions of 1 mg/mL steroid hormones were prepared in methanol. Working solutions of standards were prepared by dilution of stock solutions with methanol. Despite the fact that usage stable-isotope labeled analogs allows you to get the most accurate results, we chose methyltestosterone as internal standard because it is an exogenous steroid hormone which can also be used as a more accessible internal standard for steroid hormones (Konieczna et al., 2011). Working solutions of the derivatization agents were obtained by dissolving appropriate reagent weights in methanol to achieve 5 mg/mL concentration for HBT, 60 mg/mL concentration for AT and 20 mg/mL concentration for HBI.

Appropriate reagent weights were chosen during experiments to determine the solubility of the reagents in methanol. The working solutions of the derivatization agents were stable for a week at 4 °C. Calibration solutions containing steroid hormones at 0.015, 0.030 0.060, 0.15, 0.30, 0.60, 1.5, 3.0, 6.0, 7.5 ng/mL concentrations in synthetic urine, prepared according to (Sarigul et al., 2019), were spiked from stock solutions. Concentrations of calibration solutions were selected considering the naturally occurring levels of steroid hormones and reference intervals established in clinical diagnostic laboratories.

### 2.3 Urine samples preparation

Urine samples were obtained from male volunteers (aged between 25 and 45), which was approved by the local Ethics Committee.

Considering the established conditions, sample preparation was carried out as follows. 1 mL of acetate buffer (pH 5) containing IS and β-glucuronidase was added to 5 mL of urine, after that the mixture was incubated at 57 °C for 12 h. Then, 3 mL of carbonate buffer (pH 10), 2 g of Na_2_SO_4_ and 3 mL of diethyl ether were added, vortexed for 2 min and centrifuged for 5 min at 4,000 rpm.

The aqueous phase was frozen in cryostate and the ether layer was transferred to the tube and evaporated at 60 °C.

Then, for derivatization with HBT, evaporated layer was redissolved in 250 µL of reagent solution, 25 µL formic acid and 725 µL of methanol with further incubation at 60 °C for 120 min.

For derivatization with AT, evaporated layer was redissolved in 250 µL of reagent solution, 5 µL formic acid and 745 µL of methanol with further incubation at 70 °C for 120 min.

For derivatization with HBI, evaporated layer was redissolved in 250 µL of reagent solution, 150 µL formic acid and 600 µL of methanol with further incubation at 70 °C for 120 min.

After derivatization, mixture was evaporated under gentle nitrogen stream at 70 °C and reconstructed at 100 µL of methanol:0.1% formic acid in water (50:50) solution and transferred to the vial for further analysis.

### 2.4 Instrumentations

A Bruker MaXis Impact (Bruker Daltonik GmbH, Bremen, Germany) quadrupole-time-of-flight (Q-TOF) high-resolution mass spectrometer (HRMS) operating with an electrospray ionization (ESI) source coupled with an ultra-high performance liquid chromatography Dionex Ultimate-3000 system (UHPLC) with a Luna Omega Polar C18 (100 × 2.1 mm, 1.6 μm) column and the corresponding guard column was used in this study.

The UHPLC system consisted of the binary HPG-3200RS pump with 25 µL mixer, WPS-3000RS autosampler with thermostatting unit and TCC-3000RS column thermostat. Dionex DAD-3000RS diode-array detector was used to establish void time of the method.

The mobile phase consisted of 0.1% FA solution in acetonitrile (solvent A) and 0.1% FA in water (solvent B). Separation of analytes was conducted using gradient elution program (Table 2).

**TABLE 2:** Gradient elution program.

Time, min	Solvent A, %	Solvent B, %
0.0–0.5	2	98
0.5–14.5	100	0
14.5–17.5	100	0
17.5–17.6	2	98
17.6–23.0	2	98

The injection volume was 10 µL. The flow rate was held constant at 0.3 mL/min. During separation experiments the columns were kept at 30 °C. The voltage at the ionization source was 3.5 kV, drying gas flow rate was 8 L/min, spray gas pressure was 2 bar, temperature of the ionization source was 250 °C, mass scanning range (m/z) was 250–1,000, and scanning speed was 3 Hz. Data were collected and processed by using Bruker Compass HyStar 4.1 and Bruker Data Analysis 4.4 software, respectively.

### 2.5 Optimization of sample preparation

Sample preparation prior to analysis is one of the most critical stages, as errors at this stage typically lead to significant errors, especially in the case of multi-step procedures aimed at analyzing trace amounts of analytes in complex matrices. Derivatization is also one of these procedures, the main goal of which is to ensure the completeness of the reaction. At the same time, the reaction time should not lead to prolonged downtime for the analytical equipment and create difficulties for subsequent work. Another factor that must be taken into account is the concentration of the derivatization reagent. Typically, it is added in significant excess, which can lead to overloading of the chromatography column and, also, overloading the detector when it enters the ionization source. These circumstances necessitate the optimization of all key parameters related to the derivatization stage with particular care. Usually, optimization of sample preparation conditions is carried out by studying of the influence of one factor at a time on an experimental response, in this case one parameter is changed and others are kept at a constant level. This approach is called one-variable-at-a-time. It should be noted that this approach has several disadvantages: nonreflecting of the complete effects of the parameter on the response and increasing the number of experiments (Bezerra et al., 2014). To overcome this disadvantage, optimization of sample preparation conditions is carried out by experimental design, which allows to simultaneously optimize the studied factors (Vera Candioti et al., 2008). Taking the above into consideration, the optimization of derivatization conditions was conducted by combination of two approaches: one-variable-at-a-time and full factorial design. During optimization, a methanol solution with concentration of steroid hormones equal to 100 ng/mL was used.

Derivatives of steroid hormones were stable for 48 h, after which degradation of the samples were observed.

### 2.6 Chromatographic separation and MS detection

**FIGURE 2:**
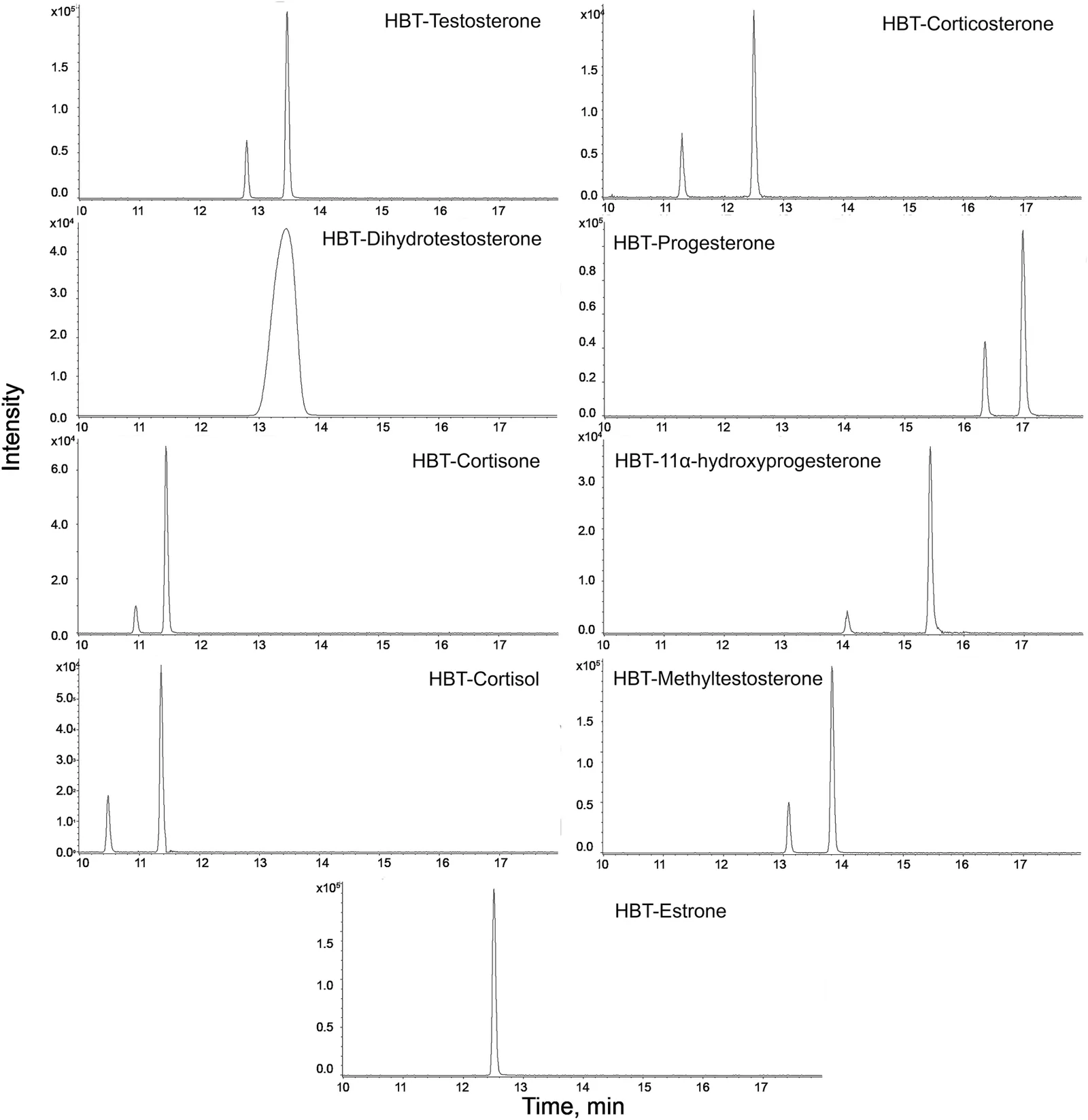
Extracted ion chromatogram of HBT-derivatives of steroid hormones in methanol solution at 100 ng/mL.

**FIGURE 3:**
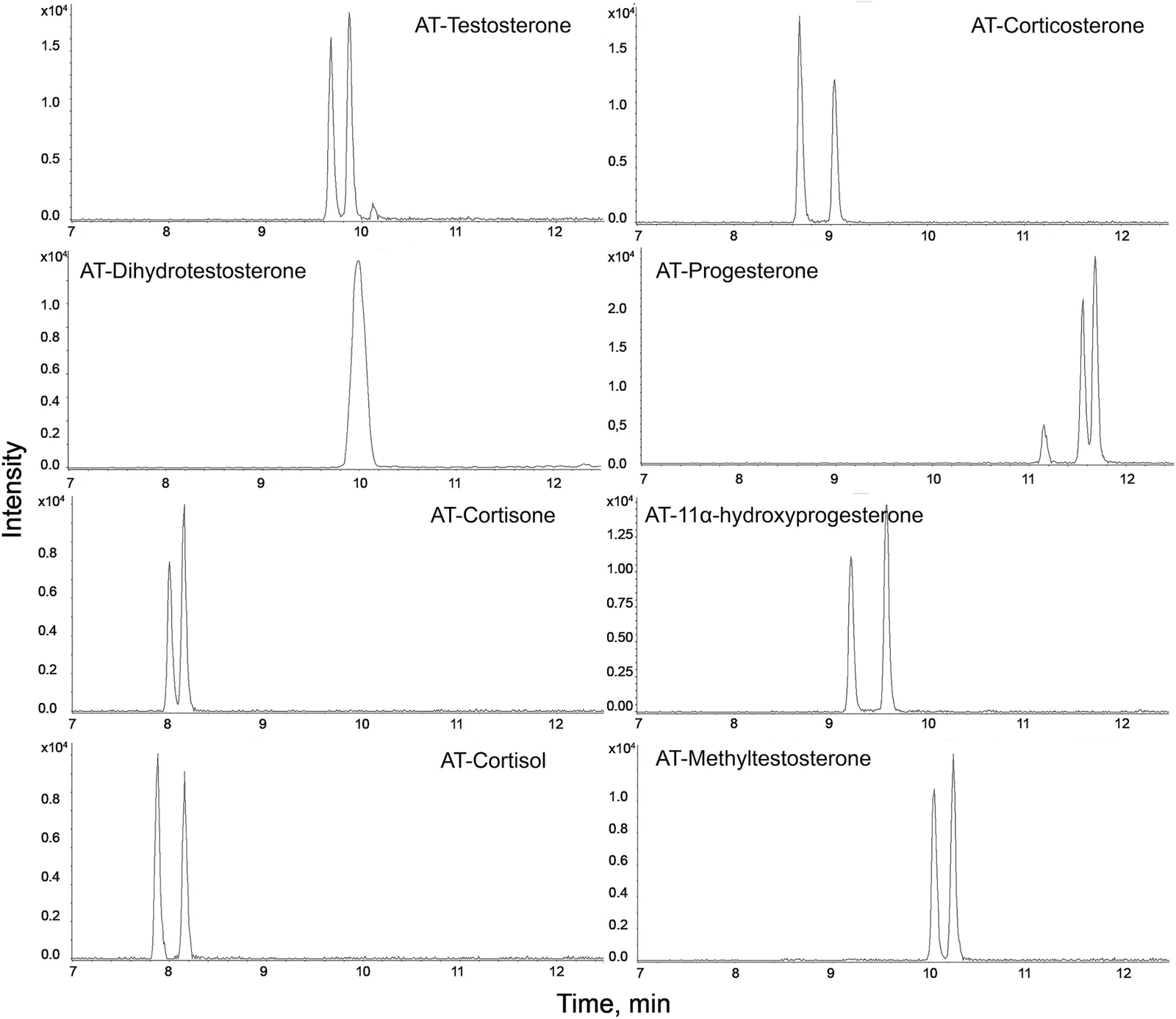
Extracted ion chromatogram of AT-derivatives of steroid hormones in methanol solution at 100 ng/mL.

**FIGURE 4:**
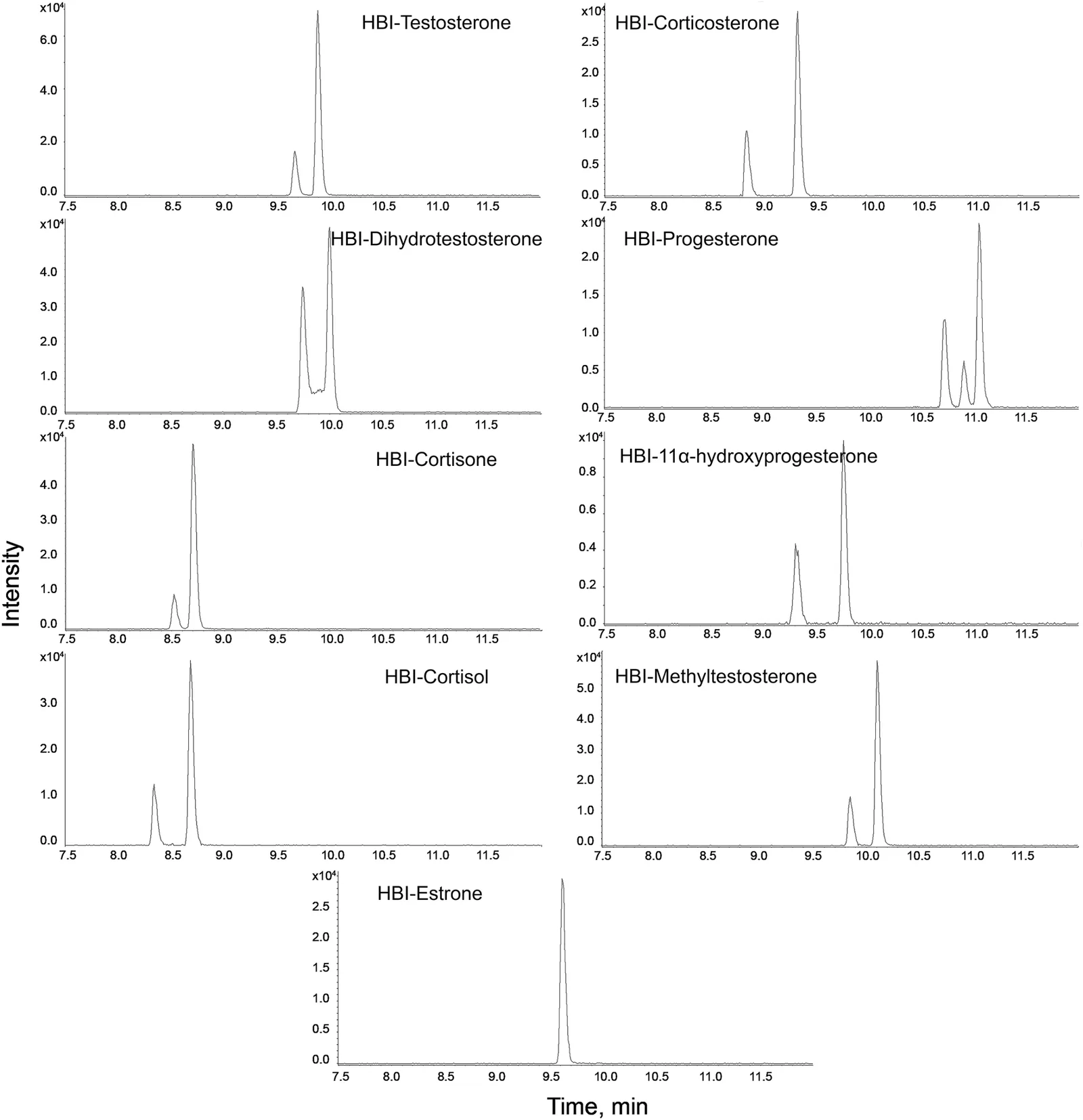
Extracted ion chromatogram of HBI-derivatives of steroid hormones in methanol solution at 100 ng/mL.

As can be seen from Figures 2–4, Supplementary Figures S1, 2, separation of studied steroid hormones derivatives was achieved with acidified acetonitrile and water gradient within 23 min. This approach can be a useful tool for separation of structurally similar steroid hormones required for routine clinical analysis. It should be noted that, to avoid any influence of the reagent on the results, it was wasted using diverter valve on the instrument for the first 3 min after injection. The column hold-up time was experimentally measured by injection of a 26 mg/mL aqueous solution of sodium nitrate and it was 0.3 min for the DAD-equipped system.

Considering that more polar functional groups are grafted during derivatization, Luna Omega Polar C18 columns provide greater separation efficiency and selectivity due to the presence of polar functional groups. Given the presence of both syn- and anti-forms in chromatograms, this is a significant aspect, especially when analyzing real samples, where enantiomers of analytes are also present.

The choice of mobile phase was determined by several factors, one of which was the need for maximum sensitivity. For the task at hand, viscosity and electrospray stability are highly significant. Unlike methanol-based mobile phases, where even hydrogen bonds play a role, an acetonitrile-based mobile phase ensures greater spray stability, and its higher elution force allows for easier control of retention parameters during gradient elution.

Acidification of the mobile phase ensures efficient proton transfer, while a salt-free environment also improves robustness and simplifies the mobile phase preparation process, which is important for instrument operators in routine analysis. The optimal formic acid concentration is 0.1%; further increases in formic acid concentration in the mobile phase lead to a drop in signal due to ionization quenching, while a decrease in concentration leads to a loss of protonation efficiency, making a concentration of 0.1% the optimal value.

## 3 Results and discussion

The development of new derivatizing agents is a modern challenge in analytical chemistry, aimed at increasing the sensitivity and informativeness of the data obtained. Although a large number of different reagents are currently known, each has its own advantages and disadvantages, as well as the need for specific detection methods. For example, FMOC and dansyl derivatives are widely known fluorescent labels, while oximes and derivatives based on Girard’s reagent P and T are widely used in mass spectrometry. However, as with oximes, they form syn- and anti-forms. At the same time, due to the presence of a fixed charge, they contribute to increased ionization efficiency. For compounds such as estrogens, this is an important advantage when it comes to their simultaneous determination with androgen components in targeted analysis. The search for new reagents capable of providing comparable or superior sensitivity to known ones is a complex and multifactorial task that requires taking into account the stability of reagents and derivatives over time, studying the reaction conditions, and also comparing metrological characteristics.

**FIGURE 5:**
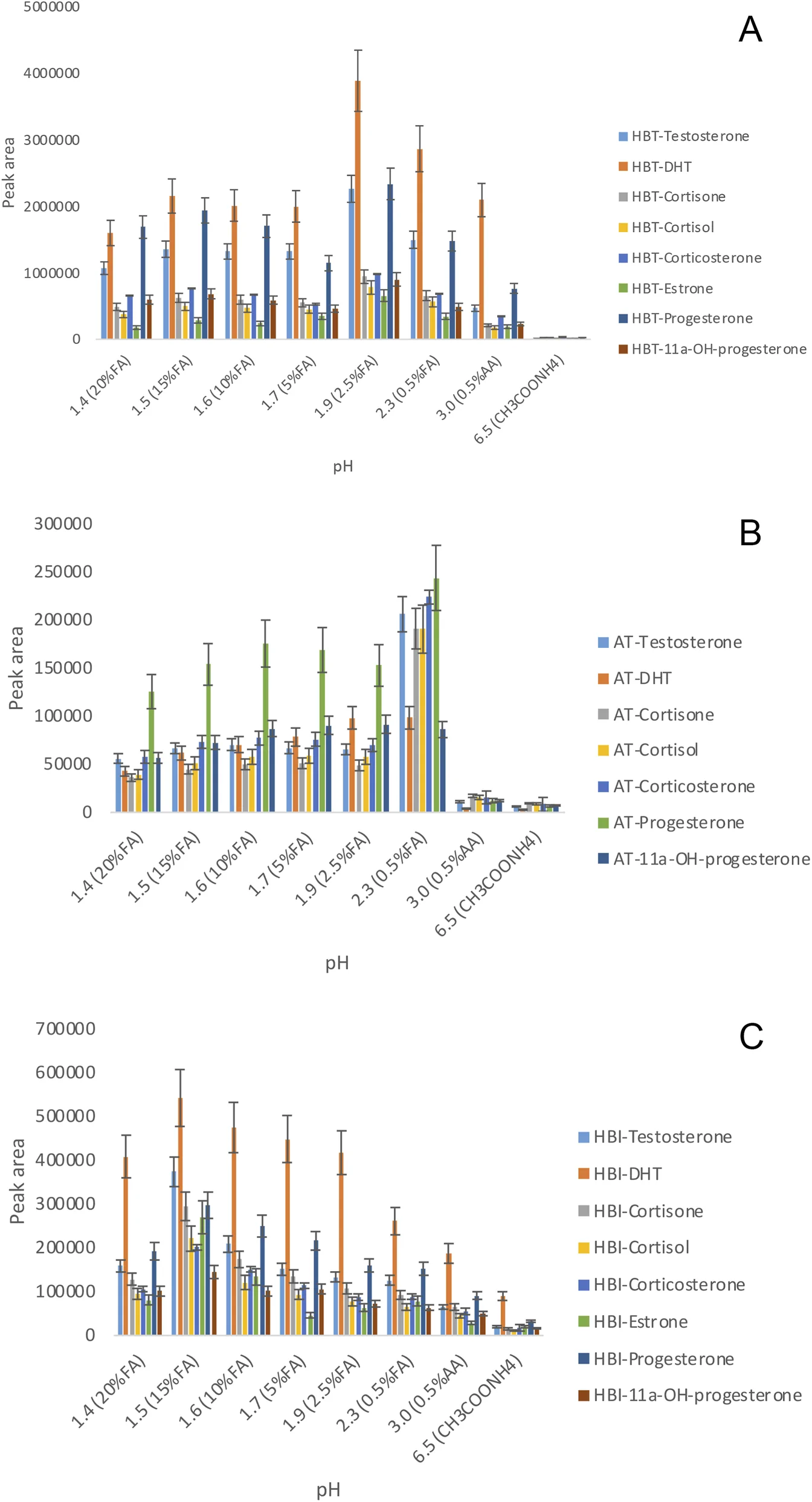
Optimization of the pH for steroid hormones derivatization with **(A)** HBT, **(B)** AT, **(C)** HBI (n = 3).

pH value is a factor that significantly influences the derivatization procedure. Since the reactions between ketosteroids and new reagents are based on the nucleophilic addition-elimination mechanism, all reactions were carried out in an acidic environment. As can be seen from Figures 5A–C, the optimal pH values for HBT, AT, HBI are 1.9, 2.3, 1.5, which corresponded to 2.5%, 0.5% and 15% formic acid concentrations in water, respectively.

**FIGURE 6:**
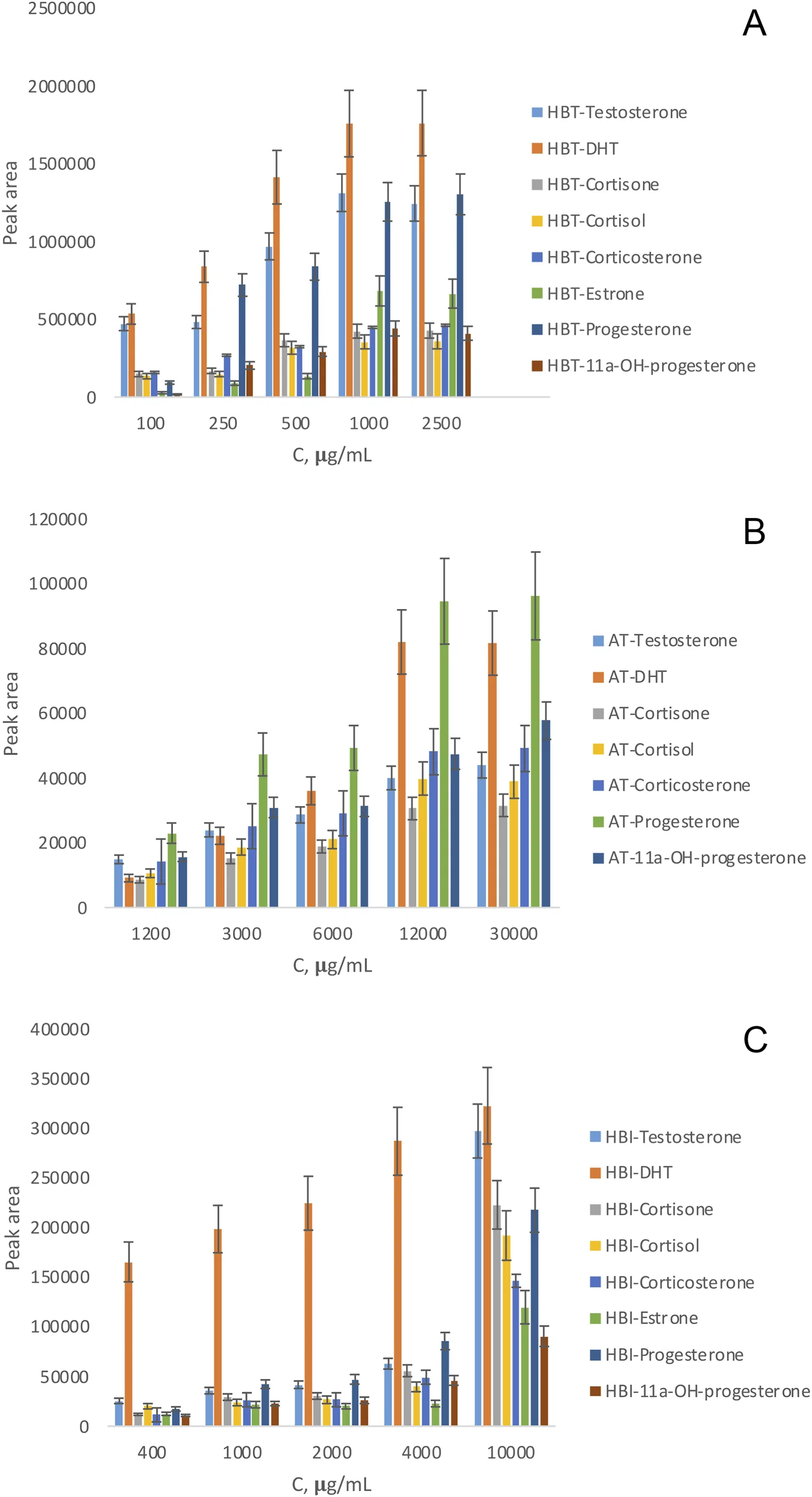
Optimization of derivatization reagent concentration **(A)** HBT, **(B)** AT, **(C)** HBI (n = 3).

The concentration of the derivatizing reagent is an equally important factor that should also be studied. The optimal concentration of HBT, AT and HBI were 1 mg/mL, 12 mg/mL and 10 mg/mL, respectively (Figures 6A–C). It should be noticed, that following increasing of the reagents concentration may lead to column saturation as well as detector. Also, it could lead to contamination of the injection port of the chromatographic system, therefore, these parameters should be optimized carefully.

The temperature and reaction time were optimized by means of full factorial design. This approach is applied to measure responses at all combinations of the factor levels (Antony, 2014). The chosen design was three level-two factors (3^2^) full factorial design consisting of nine combinations (Table 3; Table 4).

**TABLE 3:** Factor levels for full factorial experiment with HBT.

Factor	Level		
	−1	0	+1
Temperature	25	43	60
Reaction time	10	65	120

**TABLE 4:** Factor levels for full factorial experiment with AT and HBI.

Factor	Level		
	−1	0	+1
Temperature	25	48	70
Reaction time	10	65	120

**FIGURE 7:**
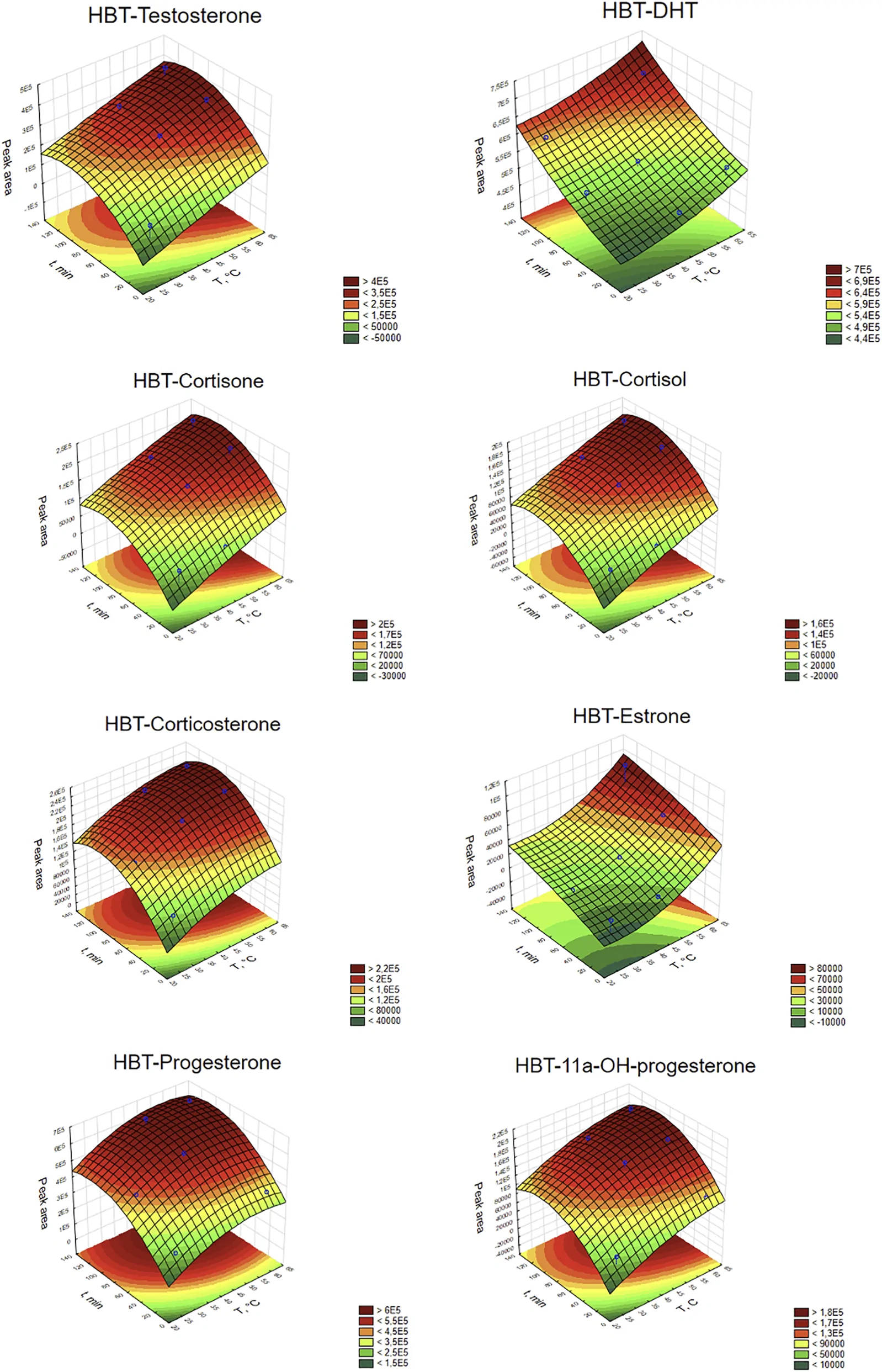
Response surfaces for temperature and time of reaction with HBT.

**FIGURE 8:**
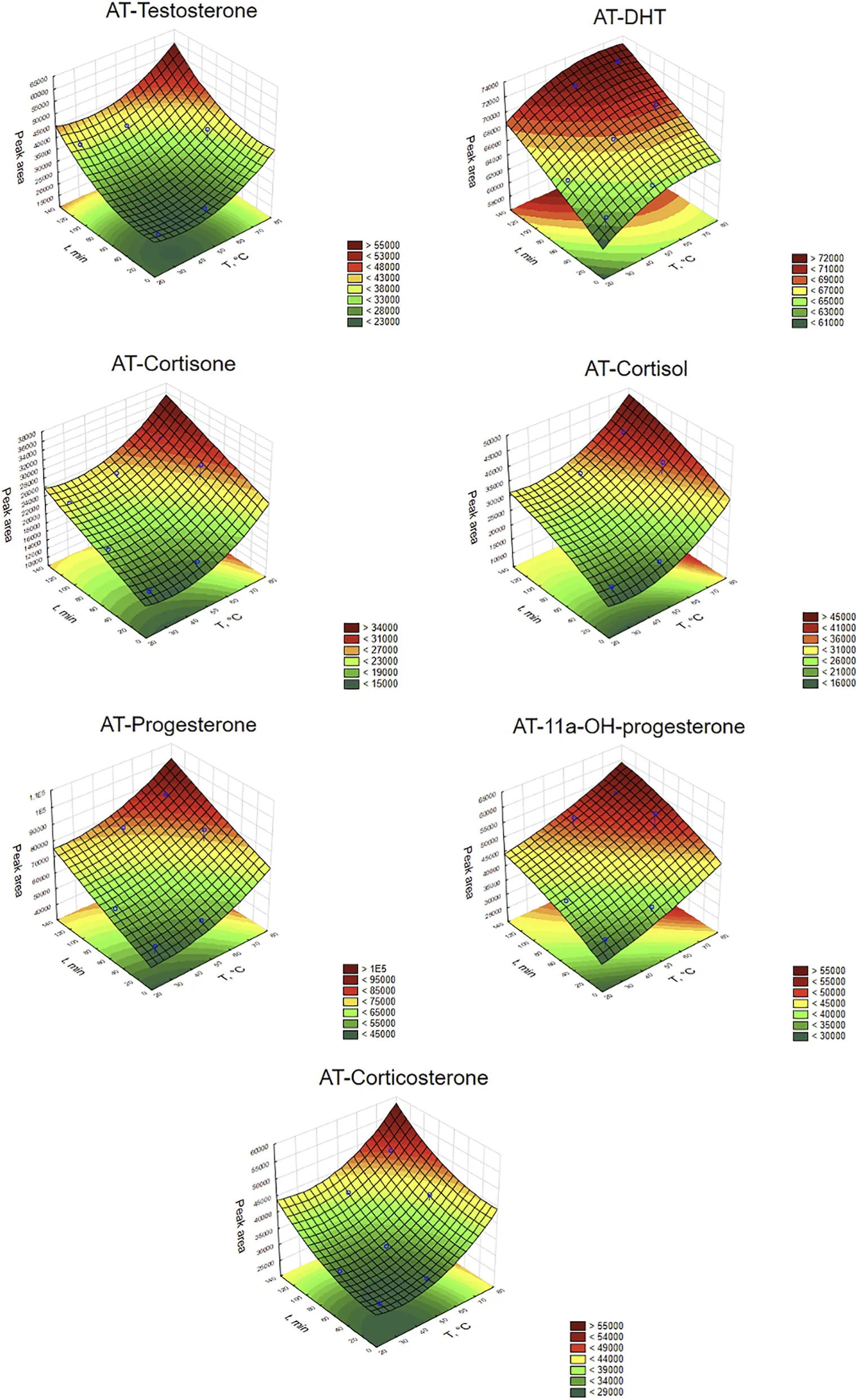
Response surfaces for temperature and time of reaction with AT.

**FIGURE 9:**
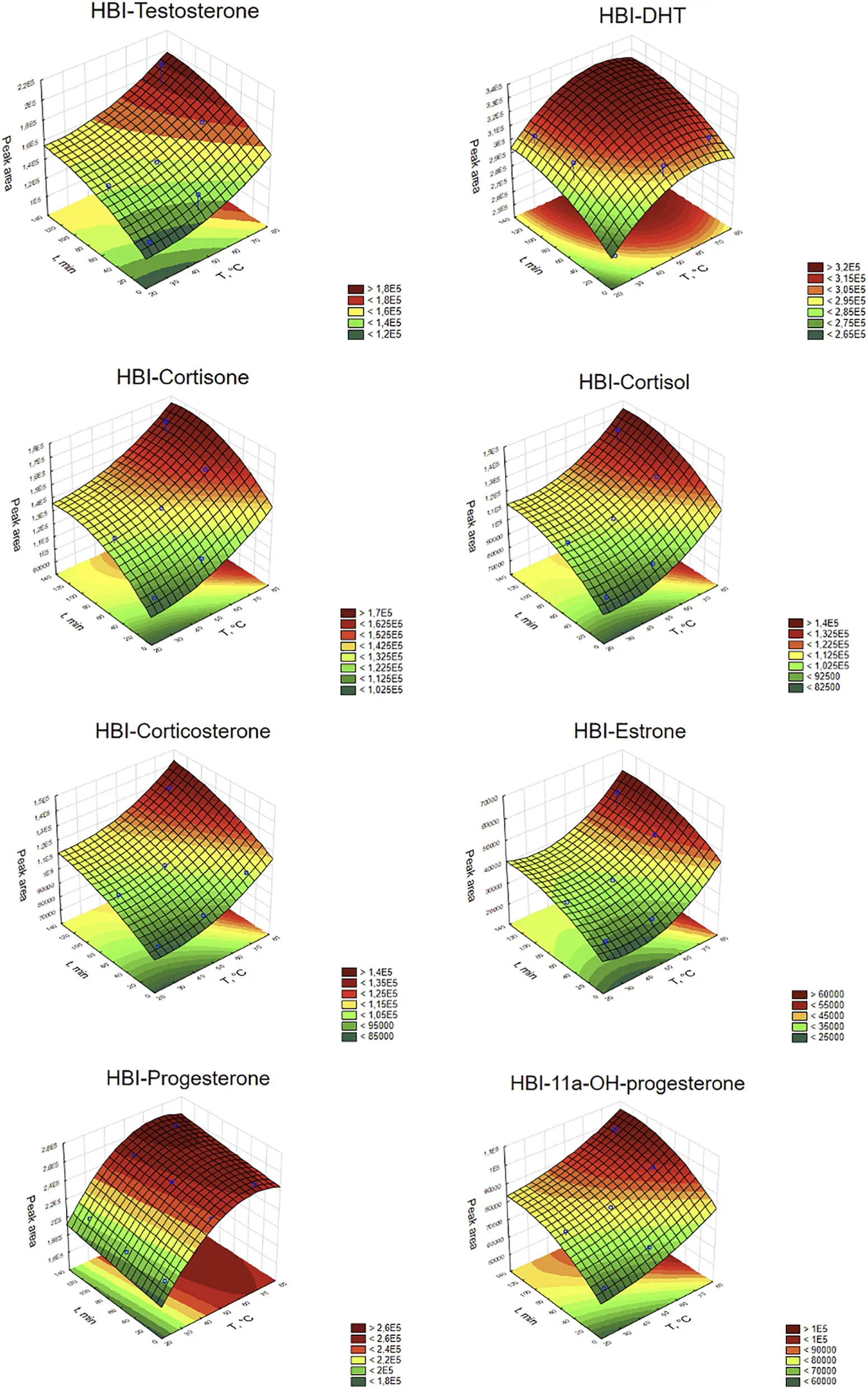
Response surfaces for temperature and time of reaction with HBI.

As can be seen from the response surfaces obtained, the best results are achieved at temperature of 60 °C and reaction time of 120 min for HBT and at temperature of 70 °C and reaction time of 120 min for AT and HBI (Figures 7–9). For some analytes, no obvious plateau was observed, but additional clarifying experiments revealed that increasing reaction time did not significantly increase peak areas and increasing temperature leads to degradation of the derivatives.

One of the key considerations in optimizing the derivatization conditions was the completeness of the reaction. Monitoring was accomplished by determining both derivatives and native compounds. Given the sensitivity of the instruments and assays used in optimization, this allows us to conclude that the reaction is truly complete, as actual endogenous concentrations are typically significantly lower. Furthermore, using high analyte concentrations allows us to partially account for reagent consumption during interactions with matrix components, which can negatively impact reaction completeness in real samples. It is important to note that the formation of syn- and anti-forms is reproducible and concentration-independent for all reagents used in this study, including hydroxylamine and Girard’s reagent T.

### 3.1 Comparison of HBT, AT and HBI with HA and GRT

As can be seen from the chromatograms presented, the new reagents behave like classical hydrazines, forming syn- and anti-forms. Similar behavior is also observed for oximes formed during interaction with hydroxylamine and for derivatives of the Girard’s reagent. In the case of HBT, AT and HBI, and the presence of forms is not equal. In this case, we took the most abundant peak for further quantitative analysis. Unfortunately, determining the correspondence of the form to each peak is a complex task and requires the preparative isolation of each component at high concentrations for further NMR analysis. Three peaks are observed for AT- and HBI-progesterone, since native progesterone contains two carbonyl groups that can be derivatized. Cortisone, cortisol and 11α-hydroxyprogesterone also contain more than one carbonyl groups that can be derivatized, but we assume that in the case of these compounds, coelution of the resulting forms is observed.

As can be seen from Table 5, the reaction with HBT was characterized by the formation of disubstituted derivatives of progesterone (Di-HBT-Progesterone) and 11α-hydroxyprogesterone (Di-HBT-11α-hydroxyprogesterone). This fact requires careful attention when optimizing sample preparation conditions using new reagents and subsequent interpretation of the results to avoid incorrect conclusions about the completeness of the derivatization reaction. It also should be noted that AT-Estrone was not formed, which may be explained by the low stability of derivative in acidic solution under high pressure conditions during chromatographic separation. The same behaviour was shown by some other 17-keto steroids, like androsterone, etiocholanolone and DHEA, which could not be detected after derivatization with AT. To confirm this hypothesis, a standard solution with AT-derivative was infused directly into the ion source, and the protonated peak of the derivative was observed.

**TABLE 5:** Comparison of chromatographic and mass-spectrometric parameters of HBT-, AT-, HBI-, HA-derivatives and native steroid hormones.

Analyte	Retention time, min	[M + H]^+^ theoretical, m/z	[M + H]^+^ observed, m/z	Mass error, ppm
HBT-Testosterone	12.8; 13.5	436.2417	436.2418	−0.23
HBT-Dihydrotestosterone	13.6	438.2574	438.2574	0.00
HBT-Cortisone	11.0; 11.5	508.2265	508.2263	0.39
HBT-Cortisol	10.5; 11.4	510.2421	510.2416	0.98
HBT-Corticosterone	11.3; 12.5	494.2472	494.2467	1.01
HBT-Estrone	12.5	418.1948	418.1946	0.48
Di-HBT-Progesterone	16.3; 17.0	609.2829	609.2837	−1.31
Di-HBT-11α-hydroxyprogesterone	14.1; 15.4	625.2778	625.2782	−0.64
HBT-Methyltestosterone	13.1; 13.8	450.2574	450.2574	0.00
AT-Testosterone	9.7; 9.9	355.2492	355.2494	−0.56
AT-Dihydrotestosterone	10.0	357.2649	357.2640	2.52
AT-Cortisone	8.0; 8.1	427.2340	427.2331	2.11
AT-Cortisol	7.9; 8.2	429.2496	429.2487	2.10
AT-Corticosterone	8.7; 9.0	413.2547	413.2546	0.24
AT-Estrone	—			
AT-Progesterone	11.2; 11.6; 11.7	381.2649	381.2647	0.52
AT-11α-hydroxyprogesterone	9.2; 9.6	397.2598	397.2594	1.01
AT-Methyltestosterone	10.0; 10.2	369.2649	369.2642	1.90
HBI-Testosterone	9.7; 9.9	419.2805	419.2800	1.19
HBI-Dihydrotestosterone	9.8; 10.0	421.2962	421.2952	2.37
HBI-Cortisone	8.5; 8.7	491.2653	491.2649	0.81
HBI-Cortisol	8.4; 8.7	493.2809	493.2799	2.03
HBI-Corticosterone	8.8; 9.3	477.2860	477.2854	1.26
HBI-Estrone	9.6	401.2336	401.2329	1.74
HBI-Progesterone	10.7; 10.9; 11.1	445.2962	445.2957	1.12
HBI-11α-hydroxyprogesterone	9.3; 9.8	461.2911	461.2904	1.52
HBI-Methyltestosterone	9.9; 10.1	433.2962	433.2957	1.15
HA-Testosterone	10.4; 10.5	304.2271	304.2267	1.23
HA-Dihydrotestosterone	11.1; 11.2	306.2428	306.2426	0.77
HA-Cortisone	8.3; 8.4	391.2227	391.2226	0.33
HA-Cortisol	8.2; 8.3	393.2384	393.2382	0.60
HA-Corticosterone	9.2; 9.3	377.2435	377.2433	0.45
HA-Estrone	10.3	286.1802	286.1797	1.64
HA-Progesterone	12.2; 12.3	345.2537	345.2527	2.91
HA-11α-hydroxyprogesterone	9.9; 10.0	361.2486	361.2476	2.77
HA-Methyltestosterone	10.7; 10.8	318.2428	318.2419	2.98
Testosterone	10.3	289.2162	289.2162	0.00
Dihydrotestosterone	11.0	291.2319	291.2319	0.00
Cortisone	8.3	361.2010	361.2010	0.00
Cortisol	8.2	363.2166	363.2168	−0.55
Corticosterone	9.2	347.2217	347.2215	0.58
Estrone	10.2	[M − H]^−^: 269.1547	[M − H]^−^: 269.1539	2.97
Progesterone	12.2	315.2319	315.2323	−1.27
11α-hydroxyprogesterone	9.7	331.2268	331.2271	−0.91
Methyltestosterone	10.7	303.2319	303.2319	0.00

As shown in Table 5, as a result of interaction between steroid hormones and new derivatizing reagents, the molecular properties of derivatives have become different from molecular properties of steroid hormones and the retention time of HBT-steroids in reversed-phase liquid chromatography mode increases compared to the retention times of native compounds, the retention time of AT-steroids and HBI-steroids, on the contrary, decreases. It is known that with an increase in the values of pKa, the basic properties of the compounds increase (Verma et al., 2019). Based on the values of the pKa of the heterocyclic compounds included in the structures of the substituents (Table 6), it can be assumed that as the ability to protonate increases, the polarity of the resulting derivatives decreases in the series HBI- > AT- > HBT-derivatives. This fact is confirmed by the value of the retention times of the obtained derivatives.

**TABLE 6:** The pKa values of the heterocyclic compounds included in the structures of the substituents.

Heterocyclic compound	pK_a_	References
Benzothiazole	7.8	Schaumann (2002)
1,2,4-Triazole	10.3	Garratt (1996)
Benzimidazole	12.8	Walba and Isensee (1961)

In comparison to conventional effective derivatizing agent such as HA, novel derivatizing agents HBT, AT and HBI contain additional nitrogen atoms, which can provide a higher ionization efficiency and improves the detection and quantification limits. This also explains the high efficiency of such reagents, like GRT, which had fixed charge in the molecule. In the case of HBT, it also contains a sulfur atom in the structure. As can be seen from Table 6, HBI have higher value of pKa than HBT. According to (Oss et al., 2010), the influence of Gas Phase Basicity, Molecular Mass of Ion, Molecular Area of Ion, Polar Surface Area of Ion, and Molecular Volume of Ion must be considered as well. As can be seen from Supplementary Table S1, in comparison with previously reported sensitivity parameters for HA (Dmitrieva et al., 2022) and GRT (Zorina et al., 2025), the highest sensitivity was achieved for HBT-derivatives, for example, LOD of HBT-estrone has decreased by 10 times compared to LOD of GRT-estrone. Taking into account the influence of the design of ionization sources on the ionization efficiency, it seemed appropriate to compare the analytical signals also obtained using a heated electrospray ionization source. To test this hypothesis, all types of derivatives were directly infused into a Thermo TSQ Quantum Access Max triple quadrupole mass spectrometer equipped with a heated ESI source ат 10 μL/min flow. The sheath gas was kept at 420 °C, the transfer capillary at 320 °C and gas flows were set to 60/10/0 a. u. (sheath/aux/sweep).

Figures 2–8 demonstrate that optimal derivatization conditions require relatively high temperatures and prolonged incubation compared to hydroxylamine. However, as shown in Supplementary Table S1, oxime derivatives do provide greater sensitivity compared to native steroid hormones, but are significantly inferior to other derivatization reagents. In targeted trace analysis, this difference can be critical, as steroid hormone concentrations can be ultra-trace, depending on gender and age.

It is important to note that the derivatization reagents under consideration demonstrated satisfactory metrological characteristics (Supplementary Table S2).

During optimization, the stability of the obtained results is an important aspect. In the case of derivatives capable of forming syn- and anti-forms in nonequivalent ratios, the reproducibility of the formation of the forms and their ratios becomes a mandatory issue. Supplementary Figure S2 shows that the concentration of formic acid present in the sample does not affect the ratio of the derivatives. This parameter was chosen for demonstration as the most significant when optimizing derivatization conditions.

Limit of detection for all compounds were established as a signal-to-noise ratio >9:1. Establishing of LLOQs for all compounds were experimental too with the acceptance criterion in this case was an error not exceeding 20%.

As it can be seen from Supplementary Table S1, for AT-derivatives LOD and LOQ are stayed constantly for all analytes. It may be caused influence of derivatization reagents, determining the efficiency of ionization of the molecule in this case.

Results of the interday and intraday accuracy and precision presented in Supplementary Table S2.

It should be noted that AT-Estrone, which is formed during the derivatization reaction (Supplementary Figure S3), is subsequently destroyed in the mobile phase flow and during passage through the column. Its stability is likely affected by both the pressure and acidity of the mobile phase, as a derivative peak was observed during direct injection of the standard sample after derivatization. This behavior of derivatives requires special attention and consideration of stability not only during storage but also during analysis, as such phenomena can significantly affect the results obtained. It is worth noting that quantitative analysis in this paper was performed using a triple quadrupole system. In addition to being the gold standard for quantitative target analysis, this system is equipped with a heated electrospray ionization source, which is more stringent and allows for more efficient ionization of analytes such as steroid hormones. In this study, this resulted in a 6- to 8-fold increase in sensitivity, depending on the type of derivative.

### 3.2 Analysis of urine samples

The analysis results of three urine samples obtained using HA, GRT and novel derivatizing agents are provided in Table 7. Urine samples were stable only for 24 h, which may be due to the presence of a significant amount of matrix components. This must be taken into account when conducting the analysis and loading the instrument, since degradation of analytes can lead to distortion of the observed results, which is especially critical when conducting clinical studies. The comparability of the results indicates the promise of further research of novel agents.

**TABLE 7:** Comparison of the urine sample analysis results.

Sample no.	Analyte	Concentration, ng/mL					
		HBT-derivatives	AT-derivatives	HBI-derivatives	HA-derivatives	GRT-derivatives	Without derivatization
1	Testosterone	6.9 ± 1.2	7.3 ± 1.3	7.4 ± 1.2	7.3 ± 1.2	7.1 ± 1.3	7.8 ± 1.5
	Dihydrotestosterone	1.4 ± 0.2	1.4 ± 0.2	1.5 ± 0.2	1.5 ± 0.2	–*	–
	Cortisone	5.6 ± 0.9	5.5 ± 0.9	5.8 ± 1.0	5.7 ± 0.9	5.8 ± 0.9	5.5 ± 0.9
	Cortisol	4.2 ± 0.7	4.3 ± 0.7	4.3 ± 0.7	4.1 ± 0.7	4.2 ± 0.7	–
	Corticosterone	3.0 ± 0.4	2.9 ± 0.4	2.8 ± 0.3	2.7 ± 0.4	2.7 ± 0.4	–
2	Testosterone	6.6 ± 1.0	6.5 ± 1.0	6.3 ± 1.1	6.7 ± 1.0	6.5 ± 1.1	6.9 ± 1.2
	Dihydrotestosterone	1.1 ± 0.2	1.2 ± 0.2	1.1 ± 0.2	1.3 ± 0.2	–	–
	Cortisone	5.8 ± 1.0	5.9 ± 1.0	6.0 ± 1.0	5.9 ± 1.0	5.7 ± 1.0	6.3 ± 1.1
	Cortisol	4.5 ± 0.7	4.5 ± 0.7	4.4 ± 0.7	4.3 ± 0.7	4.4 ± 0.7	–
	Corticosterone	3.3 ± 0.4	3.2 ± 0.4	3.3 ± 0.4	3.3 ± 0.5	3.2 ± 0.5	–
3	Testosterone	7.4 ± 1.5	7.3 ± 1.6	7.4 ± 1.5	7.1 ± 1.5	7.2 ± 1.6	8.0 ± 1.7
	Dihydrotestosterone	1.5 ± 0.2	1.6 ± 0.2	1.7 ± 0.2	1.6 ± 0.2	–	–
	Cortisone	6.3 ± 1.1	6.4 ± 1.1	6.4 ± 1.2	6.2 ± 1.1	6.5 ± 1.1	6.8 ± 1.2
	Cortisol	3.8 ± 0.5	3.9 ± 0.5	3.7 ± 0.6	3.7 ± 0.5	3.8 ± 0.5	–
	Corticosterone	2.5 ± 0.3	2.4 ± 0.3	2.4 ± 0.3	2.6 ± 0.3	2.3 ± 0.3	–

* below LOQ.

However, despite the ability to quantitatively analyze individual components, the results obtained will be limited in their informative value. Specifically, the data obtained cannot be used for non-targeted steroid screening, and the accuracy of testosterone determination will depend on the column’s ability to resolve testosterone and epitestosterone derivatives. As a result, data stability monitoring becomes paramount, increasing operator qualifications.

The absence of progesterone in the test results can be explained by the fact that only male urine samples were used to evaluate the feasibility of the proposed approach for routine use. If determination of estrogens and progestogens is required, sample preparation approaches should include significant prior sample concentration.

As can be seen from the presented data, derivatization enables quantitative analysis of a number of components that cannot be analyzed without prior concentration. For example, for native corticosteroids, as well as for DHT, insufficient detection limits were obtained, preventing their content from being assessed and compared in samples after derivatization with the same sample preparation. However, good data consistency was observed for the remaining samples.

It is important to note that for Girard T reagent and hydroxylamine, the effect of the derivatization reagent on ionization efficiency is quite significant, but is not decisive, unlike the results discussed in this study, which effectively normalized the response factors of various components.

### 3.3 Limitations of described reagents

The main issue for all reagents described in this article is the production of syn- and anti-isomeric forms of the analytes. As a result, the applicability of its usage for non-target steroidomic purposes is quite limited. A lot of potential isobaric interferences could lead to overlapping of the different compounds’ peaks and, as a result, to false results. It also should be noted that high concentrations of the derivatizion reagent should be passed to waste using the diverter valve of the instrument to exclude detector saturation and increase instrument robustness.

Another issue is optimal conditions for derivatization, which could lead to partial sample degradation in the case of analysis of such compounds, like neurotransmitters in one run, because of their oxidation. It also should be noted that for all discovered reagents, derivatization time becomes critical and could not be less described in the article for repeatability of the results.

## 4 Conclusion

This work demonstrated the applicability of HBT, AT and HBI as novel reagent for the derivatization for targeted quantitative analysis of some ketosteroids belonging to different classes. As a result of optimization of sample preparation, the maximum yield of derivatives was observed at the following conditions: for HBT – pH 1.9, 1 mg/mL concentration of reagent, 60 °C, 120 min; for AT – pH 2.3, 12 mg/mL concentration of reagent, 70 °C, 120 min; for HBI–pH 1.5, 10 mg/mL concentration of reagent, 70 °C, 120 min. The findings obtained during the study on the influence of substituents on the chromatographic characteristics of derivatives can be used to construct predictive models in the synthesis of new derivatizing reagents. A comparative study of HA, GRT and new reagents, including sensitivity parameters and results of urine samples analysis, was carried out. It was established that sensitivity parameters of procedures with HBT and HBI derivatization are similar to those of HA and GRT, which proves the applicability of the proposed procedures for the routine analysis. However, limits of detection and quantification of AT-steroids have much higher values in comparison with HA and GRT, and AT-estrone was not stable during chromatographic separation, which limits the use of this reagent for steroid profiling.

## Data Availability

The original contributions presented in the study are included in the article/Supplementary Material, further inquiries can be directed to the corresponding author.
