# Supplementary material for: An investigation of 2-hydrazinyl-1,3-benzothiazole, 4-amino-4H-1,2,4-triazole and 2-hydrazinyl-1H-benzimidazole applicability as derivatization reagents for HPLC-HRMS determination of steroid hormones in urine
Source: Front Chem. 2026 Aug 7;14:1836243. doi: 10.3389/fchem.2026.1836243 (PMC13494264; doi:10.3389/fchem.2026.1836243)
Supplement: Supplementary file 1 [file DataSheet1.docx]

Supplementary Material

**Supplementary Table 1.** Sensitivity parameters of steroid derivatives quantification (n = 6).

| **Analyte** | **HBT-derivatives** | | | **AT-derivatives** | | | **HBI-derivatives** | | | **HA-derivatives** | | | **GRT-derivatives** | | | **Without derivatization** | | |
| --- | --- | --- | --- | --- | --- | --- | --- | --- | --- | --- | --- | --- | --- | --- | --- | --- | --- | --- |
|  | **LOD, ng/mL** | **Linear range, ng/mL** | **R^2^** | **LOD, ng/mL** | **Linear range, ng/mL** | **R^2^** | **LOD, ng/mL** | **Linear range, ng/mL** | **R^2^** | **LOD, ng/mL** | **Linear range, ng/mL** | **R^2^** | **LOD, ng/mL** | **Linear range, ng/mL** | **R^2^** | **LOD, ng/mL** | **Linear range, ng/mL** | **R^2^** |
| Testosterone | 0.015 | 0.030-7.5 | 0.998 | 0.15 | 0.30-7.5 | 0.996 | 0.075 | 0.15-7.5 | 0.999 | 0.25 | 1.0–100 | 0.999 | 0.030 | 0.075-7.5 | 0.998 | 2.0 | 2–200 | 0.997 |
| Dihydrotestosterone | 0.030 | 0.075-7.5 | 0.996 | 0.15 | 0.30-7.5 | 0.999 | 0.075 | 0.15-7.5 | 0.999 | 0.25 | 1.0–100 | 0.998 | — | | | 5.0 | 5–500 | 0.995 |
| Cortisone | 0.075 | 0.15-7.5 | 0.997 | 0.15 | 0.30-7.5 | 0.996 | 0.075 | 0.15-7.5 | 0.998 | 0.5 | 2.5–100 | 0.999 | 0.075 | 0.15-7.5 | 0.997 | 2.0 | 2–200 | 0.989 |
| Cortisol | 0.075 | 0.150-7.5 | 0.996 | 0.15 | 0.30-7.5 | 0.997 | 0.075 | 0.15-7.5 | 0.997 | 0.5 | 2.5–100 | 0.996 | 0.075 | 0.15-7.5 | 0.998 | 5.0 | 5–500 | 0.991 |
| Corticosterone | 0.15 | 0.30-7.5 | 0.996 | 0.15 | 0.30-7.5 | 0.998 | 0.075 | 0.15-7.5 | 0.997 | 0.5 | 2.5–100 | 0.996 | 0.10 | 0.15-7.5 | 0.998 | 10 | 10–500 | 0.992 |
| Estrone | 0.015 | 0.030-7.5 | 0.997 | — | | | 0.075 | 0.15-7.5 | 0.998 | 0.5 | 2.5–100 | 0.999 | 0.15 | 0.30-7.5 | 0.998 | 10 | 10–500 | 0.995 |
| Progesterone | 0.015 | 0.030-7.5 | 0.999 | 0.15 | 0.30-7.5 | 0.998 | 0.15 | 0.30-7.5 | 0.998 | 0.25 | 1.0–100 | 0.999 | 0.030 | 0.075-7.5 | 0.998 | 10 | 10–500 | 0.995 |
| 11α-hydroxyprogesterone | 0.075 | 0.15-7.5 | 0.997 | 0.15 | 0.30-7.5 | 0.999 | 0.15 | 0.30-7.5 | 0.996 | 0.25 | 1.0–100 | 0.999 | 0.030 | 0.075-7.5 | 0.997 | 5 | 2–250 | 0.993 |

**Supplementary Table 2.** Results of quality control solutions analysis (n = 6).

| **Analyte** | **HBT-derivatives** | | | | | **AT-derivatives** | | | | | **HBI-derivatives** | | | | |
| --- | --- | --- | --- | --- | --- | --- | --- | --- | --- | --- | --- | --- | --- | --- | --- |
|  | **QC, ng/mL** | **Inter-day** | | **Intra-day** | | **QC, ng/mL** | **Inter-day** | | **Intra-day** | | **QC, ng/mL** | **Inter-day** | | **Intra-day** | |
|  |  | **Accuracy,**  **%** | **Precision,**  **%** | **Accuracy,**  **%** | **Precision,**  **%** |  | **Accuracy,**  **%** | **Precision,**  **%** | **Accuracy,**  **%** | **Precision,**  **%** |  | **Accuracy,**  **%** | **Precision,**  **%** | **Accuracy,**  **%** | **Precision, %** |
| Testosterone | 0.030 | -11.2 | 12.2 | -13.2 | 14.1 | 0.30 | -13.5 | 14.3 | -14.4 | 14.8 | 0.10 | -12.4 | 13.2 | -13.5 | 14.3 |
|  | 0.060 | -10.1 | 12.1 | -11.4 | 12.6 | 0.60 | -12.4 | 12.9 | -13.1 | 13.3 | 0.30 | -11.7 | 12.5 | -12.8 | 14.0 |
|  | 1.0 | 5.5 | 6.4 | 7.5 | 8.7 | 1.5 | -5.5 | 6.2 | -5.9 | 6.8 | 1.0 | -6.4 | 8.3 | -7.3 | 9.0 |
|  | 3.0 | 4.3 | 3.4 | 5.6 | 4.0 | 5.0 | -2.3 | 3.4 | -2.2 | 3.5 | 5.0 | -4.4 | 5.7 | -5.7 | 7.2 |
| Dihydrotestosterone | 0.050 | 12.4 | 12.8 | 13.1 | 14.3 | 0.30 | 12.6 | 13.1 | 12.8 | 13.0 | 0.10 | 12.8 | 13.9 | 13.6 | 14.8 |
|  | 0.10 | 8.5 | 9.7 | 9.6 | 10.3 | 0.60 | 8.8 | 9.4 | 9.2 | 9.6 | 0.30 | 9.3 | 10.2 | 10.1 | 11.5 |
|  | 1.0 | 4.6 | 5.4 | 6.0 | 6.8 | 1.5 | 4.1 | 3.3 | 5.2 | 4.0 | 1.0 | 5.8 | 6.8 | 6.9 | 8.3 |
|  | 3.0 | 2.2 | 3.4 | 2.6 | 3.4 | 5.0 | 2.9 | 2.1 | 2.9 | 2.2 | 5.0 | 3.7 | 5.0 | 4.9 | 6.2 |
| Cortisone | 0.10 | 11.4 | 13.9 | 12.8 | 13.5 | 0.30 | 10.2 | 11.0 | 11.4 | 12.5 | 0.10 | 10.4 | 11.3 | 12.0 | 12.7 |
|  | 0.20 | 6.4 | 10.5 | 7.1 | 9.2 | 0.60 | 8.2 | 8.8 | 9.3 | 10.1 | 0.30 | 9.1 | 9.8 | 10.4 | 11.7 |
|  | 1.5 | 3.4 | 4.7 | 3.3 | 4.3 | 1.5 | 5.3 | 5.9 | 5.8 | 6.4 | 1.0 | 6.2 | 7.1 | 7.3 | 7.9 |
|  | 6.0 | 2.6 | 2.9 | 2.4 | .2.9 | 5.0 | 2.5 | 3.1 | 2.8 | 3.6 | 5.0 | 3.8 | 4.5 | 4.9 | 5.6 |
| Cortisol | 0.10 | 11.9 | 12.4 | 12.6 | 14.1 | 0.30 | 12.6 | 13.6 | 13.2 | 14.2 | 0.10 | 12.3 | 12.9 | 13.2 | 13.7 |
|  | 0.20 | 7.7 | 10.7 | 7.6 | 9.0 | 0.60 | 9.9 | 10.6 | 11.0 | 12.4 | 0.30 | 10.6 | 11.2 | 10.8 | 11.6 |
|  | 1.5 | 4.9 | 6.1 | 5.4 | 6.7 | 1.5 | 6.4 | 7.3 | 6.9 | 8.1 | 1.0 | 7.9 | 8.5 | 8.8 | 9.3 |
|  | 6.0 | 2.9 | 3.5 | 3.0 | 4.1 | 5.0 | 4.3 | 5.2 | 4.7 | 5.9 | 5.0 | 5.5 | 6.1 | 6.3 | 6.8 |
| Corticosterone | 0.30 | -12.6 | 12.5 | -13.1 | 13.3 | 0.30 | -12.2 | 13.0 | -12.6 | 13.3 | 0.10 | -12.1 | 12.7 | -13.0 | 13.4 |
|  | 0.60 | -7.9 | 9.4 | -8.3 | 10.6 | 0.60 | -8.9 | 9.3 | -9.5 | 10.2 | 0.30 | -9.8 | 10.2 | -10.4 | 12.5 |
|  | 1.5 | 4.9 | 5.8 | 4.7 | 5.7 | 1.5 | -7.5 | 8.3 | -8.2 | 8.8 | 1.0 | -8.4 | 9.2 | -8.8 | 9.4 |
|  | 6.0 | 2.9 | 2.1 | 2.9 | 2.2 | 5.0 | -3.2 | 4.0 | -4.1 | 4.5 | 5.0 | -4.3 | 5.0 | -4.7 | 5.1 |
| Estrone | 0.030 | -12.3 | 13.8 | -12.2 | 13.2 | 0.30 | – | | | | 0.10 | -13.1 | 13.6 | -14.1 | 14.2 |
|  | 0.060 | -10.5 | 11.2 | -11.2 | 11.1 | 0.60 |  |  |  |  | 0.30 | -11.9 | 12.4 | -12.3 | 12.9 |
|  | 0.60 | -5.3 | 6.2 | -5.9 | 6.8 | 1.5 |  |  |  |  | 1.0 | -7.8 | 8.4 | -8.1 | 9.2 |
|  | 6.0 | -2.6 | 3.4 | -2.2 | 3.5 | 5.0 |  |  |  |  | 5.0 | -3.6 | 4.7 | -4.9 | 5.5 |
| Progesterone | 0.030 | 12.9 | 13.6 | 12.5 | 13.0 | 0.30 | 11.9 | 12.7 | 13.1 | 14.0 | 0.30 | 13.5 | 14.1 | 13.7 | 13.9 |
|  | 0.060 | 9.4 | 11.5 | 9.6 | 11.3 | 0.60 | 9.4 | 10.2 | 10.3 | 11.1 | 0.60 | 11.8 | 12.5 | 12.0 | 12.9 |
|  | 0.60 | 5.7 | 6.9 | 5.9 | 6.9 | 1.5 | 5.3 | 6.4 | 6.2 | 7.3 | 1.5 | 7.5 | 8.1 | 7.9 | 8.5 |
|  | 6.0 | 3.9 | 4.6 | 3.6 | 4.3 | 5.0 | 2.8 | 3.8 | 3.9 | 4.7 | 5.0 | 4.8 | 5.2 | 4.9 | 5.5 |
| 11α-Hydroxyprogesterone | 0.10 | 12.6 | 13.9 | 12.9 | 13.4 | 0.30 | 12.2 | 13.2 | 13.2 | 13.7 | 0.30 | 12.7 | 13.2 | 13.5 | 13.9 |
|  | 0.20 | 9.5 | 11.8 | 9.6 | 11.6 | 0.60 | 9.2 | 10.4 | 9.7 | 11.0 | 0.60 | 10.6 | 11.3 | 11.8 | 12.5 |
|  | 0.60 | 4.5 | 5.4 | 4.6 | 4.6 | 1.5 | 5.5 | 6.2 | 6.8 | 7.6 | 1.5 | 6.6 | 7.0 | 7.6 | 8.0 |
|  | 6.0 | 3.9 | 4.7 | 3.6 | 4.3 | 5.0 | 3.1 | 4.7 | 4.3 | 5.5 | 5.0 | 4.7 | 5.3 | 5.1 | 5.6 |

**Supplementary Figure 1.** Extracted ion chromatogram of HBT-, AT- and HBI- derivatives of steroid hormones in methanol solution at LLOQ.

HBT-derivatives

AT-derivatives

HBI-derivatives

**Supplementary Figure 2.** High resolution mass spectra of AT-Estrone after direct injection

**Supplementary Figure 3.** Extracted ion current for testosterone derivatives (A – AT-; B – HBI-; C – HBT-), produced at different concentrations of formic acid (2.5; 5; 10% correspondently) (n = 3)
